# Review of the Synthesis and Degradation Mechanisms of Some Biodegradable Polymers in Natural Environments

**DOI:** 10.3390/polym17010066

**Published:** 2024-12-30

**Authors:** Xiao Yao, Xue Yang, Yisang Lu, Yinyuan Qiu, Qinda Zeng

**Affiliations:** 1 School of Ecological Environment and Urban Construction, Fujian University of Technology, Fuzhou 350118, China; 18041778r@connect.polyu.hk (X.Y.);; 2 School of Mechanical and Automotive Engineering, Fujian University of Technology, Fuzhou 350118, China; 3 Fujian Special Equipment Inspection and Research Institute, Fuzhou 350008, China

**Keywords:** biodegradable polymers, polymer degradation, biodegradable lactic acid polymers, biodegradable starch-based polymers, plant fiber-based polymers

## Abstract

The escalating demand for sustainable materials has been fueling the rapid proliferation of the biopolymer market. Biodegradable polymers within natural habitats predominantly undergo degradation mediated by microorganisms. These microorganisms secrete enzymes that cleave long-chain polymers into smaller fragments for metabolic assimilation. This review is centered around dissecting the degradation mechanisms of specific biodegradable polymers, namely PLA, starch-based polymers, and plant fiber-based polymers. Recent investigations have unveiled that PLA exhibits augmented biocompatibility when combined with HA, and its degradation is subject to the influence of enzymatic and abiotic determinants. In the case of starch-based polymers, chemical or physical modifications can modulate their degradation kinetics, as evidenced by Wang et al.’s superhydrophobic starch-based nanocomposite cryogel. For plant fiber-based polymers, the effects of temperature, humidity, and cellulose degradation on their properties, along with the implications of various treatments and additives, are probed, as exemplified by Liu et al.’s study on jute/SiO_2_/PP composites. Specifically, with respect to PLA, the polymerization process and the role of catalysts such as SnCl_2_ in governing the structure and biodegradability are expounded in detail. The degradation of PLA in SBF and its interaction with β-TCP particles constitute crucial aspects. For starch-based polymers, the enzymatic degradation catalyzed by amylase and glucosidase and the environmental impacts of temperature and humidity, in addition to the structural ramifications of amylose and amylopectin, are further elucidated. In plant fiber-based polymers, the biodegradation of cellulose and the effects of plasma treatment, electron beam irradiation, nanoparticles, and crosslinking agents on water resistance and stability are explicated with experimental substantiation. This manuscript also delineates technological accomplishments. PLA incorporated with HA demonstrates enhanced biocompatibility and finds utility in drug delivery systems. Starch-based polymers can be engineered for tailored degradation. Plant fiber-based polymers acquire water resistance and durability through specific treatments or the addition of nanoparticles, thereby widening their application spectrum. Synthetic and surface modification methodologies can be harnessed to optimize these materials. This paper also consolidates reaction conditions, research techniques, their merits, and demerits and delves into the biodegradation reaction mechanisms of these polymers. A comprehensive understanding of these degradation mechanisms is conducive to their application and progression in the context of sustainable development and environmental conservation.

## 1. Introduction

Polymers play a key role in various aspects of daily life, and recent years have witnessed a growing emphasis on their environmental friendliness and sustainability. For instance, PLA has attracted extensive attention due to its potential to reduce plastic pollution in the packaging industry and its application in biodegradable medical devices. Studies by the authors of [1,2,3] have demonstrated its practical uses. Polyhydroxyalkanoates (PHAs) possess unique biodegradability and biocompatibility properties, making them suitable for various biomedical and environmental applications. Starch-based polymers, with their high biodegradability and potential for modification, have been investigated in numerous studies. Synthesizing polymers from waste, renewable resources, or recycled materials can optimize resource utilization and reduce reliance on fresh raw materials. A polymer that can remain functional within the biological or technological cycles of a circular economy throughout its lifespan is considered sustainable [4]. A polymer that degrades into harmless products in the natural environment at the end of its life is deemed environmentally friendly [5]. A detailed discussion on the variety of reaction conditions across different studies is included, providing a comprehensive overview that reflects the complexity and diversity of current research in the field of biodegradable polymers. Moreover, the environmental implications of these synthesis processes are critically analyzed to provide a holistic view of sustainability in polymer production.

Polymers are ubiquitously present in natural and synthetic realms [6]. Recent research breakthroughs have significantly broadened the domain of biodegradable polymers. For instance, Wang et al. [7] developed a superhydrophobic starch-based nanocomposite cryogel, which shows great promise in oil removal and environmental protection applications. These polymers are now intensively explored for their environmental advantages and degradation pathways [8]. For example, polylactic acid (PLA) has been widely used in packaging and biomedical fields due to its biodegradability and biocompatibility. Starch-based polymers have shown great potential in food packaging and environmental remediation with the help of specific modifications. Plant fiber-based polymers are increasingly applied in various industries because of their renewable and biodegradable properties. These polymers are particularly notable for their applications in areas ranging from agricultural films to biomedical devices, where degradation can be precisely controlled and utilized to minimize their environmental impact [9]. The unique properties of polymers make them ideal for numerous applications. For instance, polymers with low density and high tensile strength are used to manufacture lightweight products, while those with chemical stability and heat resistance are employed in harsh environments requiring durability [10]. Generally, polymers are synthesized via polymerization reactions, in which small monomer molecules chemically bond to form long polymer chains [11]. These polymerization reactions typically require catalysts and specific conditions to proceed efficiently. Condensation reactions, which link monomers and release a small molecule like water, are commonly adopted to synthesize biopolymers [12]. Bio-based polymers are generally regarded as more environmentally friendly than oil-based ones; however, the global warming potential and environmental impacts of these polymers can vary widely depending on the specific type [13]. Bio-based polymers are derived from renewable biological materials, such as plants and microorganisms, whereas oil-based polymers are sourced from non-renewable fossil fuels like petroleum [14]. Compared to oil-based polymers, bio-based ones typically consume less energy and water, suppress greenhouse gas emissions, and reduce reliance on finite resources, thus supporting the development of a circular economy [15]. Hence, biopolymers are often regarded as more sustainable alternatives to oil-based polymers. This review comprehensively analyzes the degradation mechanisms of specific biodegradable polymers, which is essential for their environmental applications and sustainability.

**Figure 1:**
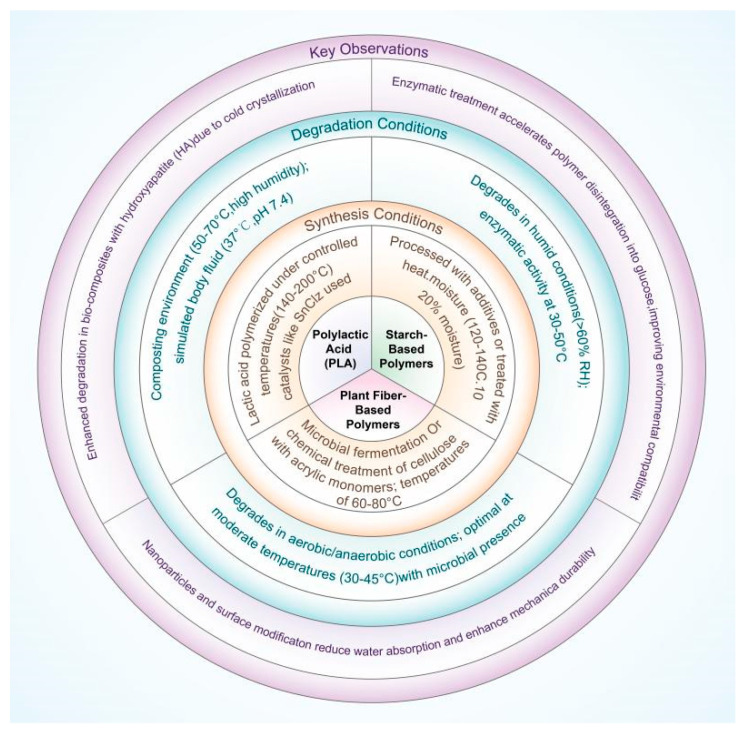
The degradation mechanisms of the three classes of bio-based polymers.

This review comprehensively analyzes the degradation mechanisms of specific biodegradable polymers, which is essential for their environmental applications and sustainability. It provides a detailed understanding of the synthesis and degradation conditions, filling the gap in current research and offering valuable insights distinct from other publications. The in-depth exploration of the interaction between polylactic acid and hydroxyapatite, the unique properties of starch-based polymers with specific modifications, and the degradation process of plant fiber-based polymers under various conditions are the key focuses that set this review apart, providing crucial knowledge for the scientific community to further advance the field of biodegradable polymers and contribute to sustainable development. Figure 1 presents the degradation mechanisms of the three classes of bio-based polymers, which are crucial for understanding their environmental fate. The degradation of PLA is initiated by the hydrolysis of its ester bonds, and this process can be accelerated by factors such as elevated temperature and humidity, as well as the presence of specific enzymes. Starch-based polymers degrade mainly through the cleavage of α-1,4-glycosidic linkages by amylase and glucosidase, and their degradation is also affected by environmental conditions. Plant fiber-based polymers degrade via the biodegradation of cellulose, which is influenced by temperature, humidity, and the activity of cellulase in fungi and bacteria.

## 2. Bio-Based and Biodegradable Polymers

### 2.1. Overview

The synthesis of bio-based polymers often involves specific reaction conditions, including temperature, pressure, and catalysts, which are crucial for optimizing polymer properties. An extended review of polymerization, fermentation, and chemical modification techniques is presented, citing a wider range of studies to illustrate the varied approaches and their environmental impacts. For instance, polymerization can offer high control over molecular weight but may require stringent conditions and catalysts. Bio-based polymers are derived from renewable materials, primarily biomass [16]. Throughout the 20th century, the polymer industry encountered challenges such as global warming and the depletion of crude oil resources [17]. Consequently, in recent years, extensive research efforts have been dedicated toward bio-based polymers, which demonstrate the potential to reduce reliance on finite fossil resources and lower carbon dioxide emissions responsible for global warming [10]. A viable approach to addressing these issues is synthesizing polymers from sustainable resources rather than fossil-fuel-derived components [18,19]. For instance, agricultural waste derived from crops such as corn or potatoes can serve as a valuable resource for producing various biopolymers [20]. In recent years, researchers and industry professionals have dedicated tremendous efforts to protect land used for food production while advancing new biotechnologies and green chemical methods [21].

**Figure 2:**
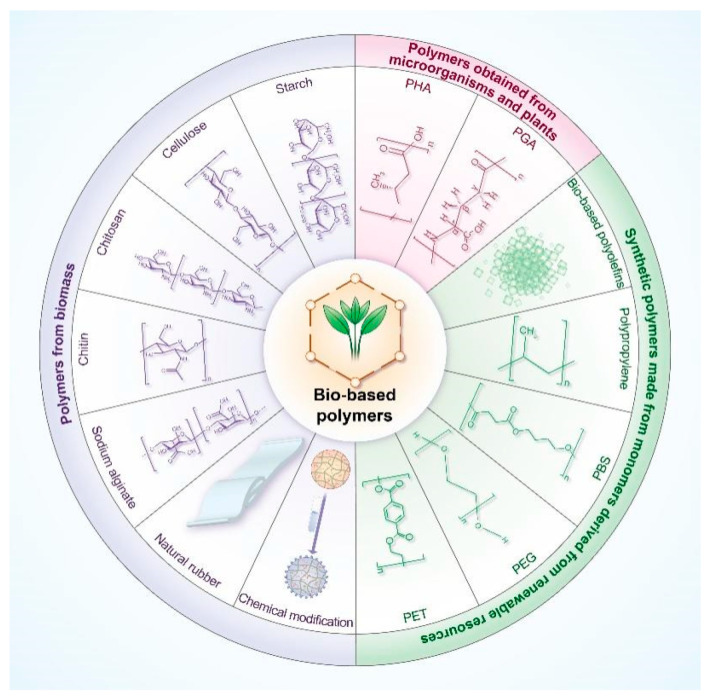
Three primary classifications of bio-based polymers.

As depicted in Figure 2, bio-based polymers can be categorized into three primary groups: (i) polymers derived from biomass, such as starch, cellulose, chitosan, chitin, sodium alginate, and natural rubber, as well as chemically modified polymers [22]; (ii) polymers sourced from microorganisms and plants, such as polyhydroxyalkanoates and polyglutamic acid [23]; and (iii) synthetic polymers produced from renewable-resource-derived monomers, such as bio-based polyolefins, polypropylene (PP), polybutylene succinate, polyethylene glycol ester, and polyethylene terephthalate (PET) [24]. In addition to these sustainable sources, the entire polymer value chain, including material design and recycling, should align with the principles of the circular economy [24].

Despite the rapid expansion of the biopolymer market in recent years, biopolymer production remains limited [25]. For instance, approximately 2.4 million tonnes of biopolymers are anticipated to be produced in 2024, representing only 1% of the total polymer production. The primary biodegradable bio-based polymers include starch and starch-based polymers, polylactide (PLA), and polybutylene adipate terephthalate [14]. In contrast, non-biodegradable bio-based polymers include polyethylene (PE), polyamide (PA), PET, and polytrimethylene terephthalate [26]. Therefore, with the growing demand for sustainable materials, the biopolymer market has considerable potential for expansion.

Non-biodegradable substances do not break down in the natural environment, posing a potential environmental threat [27]. Despite this, biodegradable biopolymers account for only 55.5% of total global biopolymer production [28]. Microorganisms can break down biopolymers under appropriate conditions without harming the environment [29]. The abovementioned percentage highlights the rising global importance of sustainable materials and increased awareness and actions toward reducing plastic pollution [28]. However, technological applications and industrial practices often require non-biodegradable bio-based polymers owing to their superior durability, stability, and suitability for long-term use under various environmental conditions [14]. Additionally, environmental factors, such as temperature, pH, and microbial population dynamics, play critical roles in determining the degradation efficiency of bio-based polymers, highlighting the need for detailed mechanistic studies. To provide a more detailed understanding of the synthesis and degradation conditions of biodegradable polymers discussed in this paper, we summarize key experimental conditions involving polylactic acid (PLA), starch-based polymers, and plant fiber-based polymers. Table 1 details the conditions under which these polymers are synthesized, the conditions under which they are degraded, and the main observations under specific circumstances. This information is essential for evaluating the use of these materials in sustainable practices.

### 2.2. PLA

Owing to the sustainability and eco-friendly properties of PLA, numerous businesses and consumers are opting for PLA-based products [1]. Polylactic acid can degrade through multiple mechanisms. Hydrolytic degradation occurs when the ester bonds in PLA are cleaved by water molecules. In a study by Zaaba et al. [38], it was found that the hydrolysis rate of PLA was significantly affected by temperature and humidity. For instance, at temperatures above 50 °C and humidity exceeding 90%, the hydrolysis rate of PLA could be accelerated by approximately 30–50% compared to normal environmental conditions. The presence of catalysts, such as tin(II) chloride (SnCl_2_), could further enhance the hydrolysis process. SnCl_2_ can act as a Lewis acid catalyst, facilitating the cleavage of ester bonds. Under experimental conditions with 0.5% (by weight) of SnCl_2_ added the hydrolysis rate of PLA was observed to increase by about 40% compared to the pure PLA sample at the same temperature and humidity. Photodegradation is another possible pathway, where exposure to ultraviolet light can cause chain scission and alteration of the polymer structure. Microbial degradation involves the action of microorganisms, such as bacteria and fungi, which secrete enzymes capable of breaking down PLA. Enzymatic degradation, specifically, can be mediated by enzymes like esterases, proteases, and lipases. These enzymes can recognize and hydrolyze the ester bonds in PLA, leading to its degradation into smaller fragments. The specific activity and effectiveness of these enzymes depend on various factors, including the enzyme source, environmental conditions, and the structure of PLA itself. For example, lipases from Pseudomonas species have been shown to be highly effective in degrading PLA, with a degradation rate of up to 0.05–0.1 mg/hour under optimal conditions of pH 7–8 and a temperature of 37 °C. In a recent study by Omigbodun et al. [39], a new type of PLA composite incorporated with nano-hydroxyapatite was developed. The addition of nano-hydroxyapatite not only enhanced the mechanical properties of PLA but also regulated its degradation rate. Through in-vitro degradation tests, it was observed that the composite exhibited a more stable and predictable degradation profile compared to pure PLA. This was attributed to the interaction between the nano-hydroxyapatite and PLA matrix, which affected the hydrolysis process of PLA ester bonds. The research provided valuable insights for the application of PLA-based materials in tissue engineering, such as in the development of biodegradable scaffolds. For instance, in the packaging industry, PLA is used to manufacture disposable tableware, plastic bags, and other products, effectively mitigating the adverse effects of plastic pollution [2]. Meanwhile, in the medical field, PLA is used to manufacture sutures and stents, reducing the need for secondary surgery owing to its biodegradability [3]. Therefore, PLA is often considered an ideal alternative to conventional plastics owing to its sustainable and biodegradable properties. Monomeric lactic acid is a natural organic acid produced through fermentation or chemical synthesis [40]. During the polymerization of lactic acid, carboxyl groups from the monomer molecules bond with hydroxyl groups [41]. The ester bonds linking individual lactic acid monomers result in the formation of polymer chains. By controlling the number of monomers and polymerization conditions, two types of lactic acid polymers can be synthesized: PLA and polylactide hydroxyacid.

**Figure 3:**
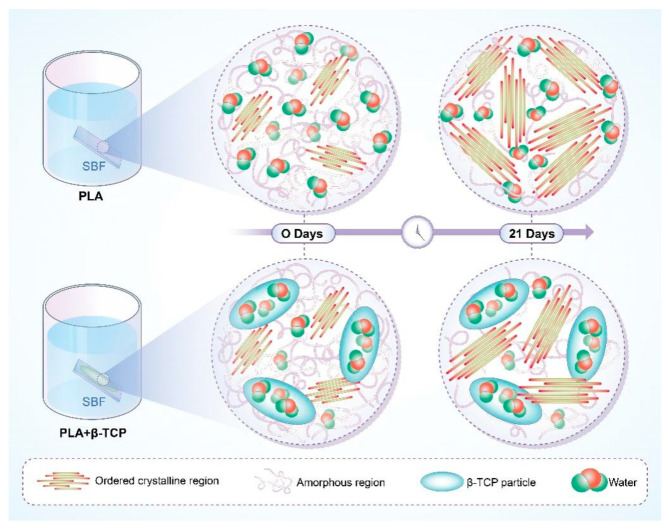
Mechanism of the PLA-mediated degradation of β-TCP particles [42].

The reaction mechanism of PLA involves the polymerization of lactic acid under controlled conditions, which are detailed in terms of the temperature and catalysts used. The advantages of using specific catalysts like SnCl_2_ include enhanced control over the polymer structure, which is critical for applications requiring precise biodegradability. Baino et al. [42] conducted an experiment where PLA was immersed in simulated body fluid (SBF). In this process, the interaction between PLA and the components in SBF led to the formation of particles within the polymer matrix. These particles were mainly formed due to the ion exchange and reaction between PLA and substances like β-TCP in SBF. The calcium and phosphate ions in SBF interacted with the β-TCP particles and gradually infiltrated into them, inducing surface recombination and dissolution reactions. Over time, a loose Ca–P framework was formed on the particle surfaces as calcium and phosphorus ions from SBF were deposited. This series of reactions not only changed the chemical composition and morphology of β-TCP but also affected the properties of the PLA composite, ultimately leading to the formation of particles within the polymer matrix. This led to a significant enhancement in PLA degradation through a cold crystallization process. The detailed mechanism of this interaction, which involves the reaction between PLA and β-TCP particles, is vividly illustrated in Figure 3. Specifically, the ion exchange method was employed to synthesize an HA-PLA composite. The study found that SBF reacted more effectively with the HA-PLA composite than with β-tricalcium phosphate (TCP) alone. This interaction remarkably improved both the mechanical properties and biocompatibility of the material. Another investigation by Ftiti [32] revealed that immersing β-TCP in SBF induced changes in its density, morphology, and chemical composition. Furthermore, research by Sirianni et al. [43] has shown that the addition of certain fillers or modifiers to PLA can also influence its degradation behavior.For example, the incorporation of nanoparticles can affect the diffusion of water and oxygen within the polymer matrix, thereby altering the rate and mechanism of degradation. When adding 5% (by weight) of silica nanoparticles to PLA, the water diffusion coefficient decreased by approximately 20–30%, leading to a slower hydrolytic degradation rate. In the early stages of soaking, the calcium and phosphate ions in the SBF interacted with each other and with the β-TCP particles. Over time, these ions infiltrated the interior of the β-TCP particles and interacted with the β-TCP surface, inducing surface recombination and dissolution reactions.In the early stages of soaking, the calcium and phosphate ions in the SBF interacted with each other and with the β-TCP particles. Over time, these ions infiltrated the interior of the β-TCP particles and interacted with the β-TCP surface, inducing surface recombination and dissolution reactions. Furthermore, through ion exchange and diffusion reactions, calcium and phosphorus ions from the SBF gradually deposited on the surfaces of β-TCP particles to form a loose Ca–P framework. Meanwhile, the phosphate ions within the β-TCP particles gradually dissolved and were substituted by phosphate ions from the SBF [32]. During the intermediate stage of this transformation, the Ca–P layer on the surface of the particles continually expanded and gradually became denser. Although the study by [32] was centered around the in vitro characterization of a covalently photo-crosslinked polymer/bioactive glass hybrid for bone tissue engineering and did not directly address our core biodegradable polymers, its findings on the immersion of β-TCP in SBF and the resultant changes in density, morphology, and chemical composition can offer a comparative perspective. The alterations in β-TCP properties due to SBF exposure, such as ion exchange and surface recombination reactions, share similarities with the processes involved in the degradation of some biodegradable polymers. For instance, in the context of PLA and β-TCP composites, the ion exchange and surface reactions play a crucial role in the degradation mechanism. By understanding the behavior of β-TCP in a different polymeric system, we can potentially draw parallels and gain a more comprehensive understanding of the complex interactions that occur during the degradation of biodegradable polymers, especially when considering their biomedical applications. This knowledge can contribute to the broader understanding of how different components within a composite material can influence its overall performance and degradation characteristics, which is pertinent to our exploration of the degradation mechanisms and applications of biodegradable polymers.

Rakmae et al. [44] carried out a study that focused on the impact of PLA’s molecular weight on the distribution of HA particles within the polymer matrix. Their results showed that due to the strong and stable interface between the PLA and HA particles, the buffer solution had great difficulty in penetrating the composite material. This characteristic is of particular importance in applications such as drug delivery, where reliable and controlled methods for drug storage and release are essential. The authors emphasized that the incorporation of HA particles opens up novel avenues for PLA applications and provides valuable insights for future research in composite material development. They also anticipated that future studies would further clarify the interaction mechanisms within PLA–HA composite materials and examine the effects of these interactions on polymer properties. Consequently, the HA particles effectively inhibited solution penetration and preserved the integrity of the composite material. The authors highlighted the importance of this stability in applications such as drug delivery, which require reliable and controlled methods for drug storage and release. They underscored that the incorporation of HA particles opens up new avenues for PLA applications and provides a valuable reference for future research in composite material development. Moreover, the researchers anticipated that future studies would clarify the interaction mechanisms within PLA–HA composite materials and examine the effects of these interactions on polymer properties.

In addition, the degradation mechanism of PLA was also emphasized, which mainly involves the hydrolysis of its ester bond. In the natural environment, when the temperature reaches above 50 °C, and the humidity exceeds 90%, the degradation rate of PLA is significantly accelerated [45]. In particular, enzymes such as lipases and proteases have been shown to effectively accelerate the degradation process of PLA. These findings are critical for designing PLA applications for controlled degradation [46].

The mechanism shown in Figure 3 illustrates how PLA interacts with β-TCP particles in the presence of simulated body fluid (SBF). As the PLA is immersed in SBF, the ion exchange between the PLA and β-TCP particles initiates a series of reactions. The calcium and phosphate ions in the SBF start to interact with the β-TCP particles and gradually infiltrate into them. This leads to surface recombination and dissolution reactions on the β-TCP particles. Over time, a loose Ca–P framework is formed on the particle surfaces as calcium and phosphorus ions from the SBF deposit. Meanwhile, the phosphate ions within the β-TCP particles dissolve and are replaced. This process not only changes the chemical composition and morphology of β-TCP but also significantly affects the properties of the PLA composite. The enhanced degradation of the bio-composite with hydroxyapatite (HA) due to cold crystallization is a crucial aspect. The presence of HA promotes the formation of crystalline regions within the composite during cold crystallization. These crystalline regions have different physical and chemical properties compared to the amorphous regions. They are more susceptible to hydrolysis and enzymatic attack, which accelerates the overall degradation process of the composite. The specific interaction between the HA particles and the polymer matrix is complex. The HA particles can act as nucleation sites for crystallization, and they also influence the diffusion of water and enzymes within the matrix. This leads to a more efficient degradation pathway, making the composite more suitable for applications where controlled degradation is required, such as in drug delivery systems.

### 2.3. Starch-Based Polymers

Starch-based polymers are polymers with starch as a principal constituent. Starch, a natural polysaccharide composed of glucose monomers, can be combined with other substances or chemically modified to synthesize starch-based polymers. For example, MaterBi, a renowned starch-based material, is synthesized by blending starch with other biodegradable polymers and additives. As per Aldas et al.’s study [47], MaterBi showcases good biodegradability and mechanical properties. It can be fabricated into diverse forms like films and fibers, finding potential applications in packaging and biomedical arenas. Its degradation mainly occurs via the enzymatic hydrolysis of starch by amylase and other environmental enzymes, and the presence of other components in MaterBi can influence its degradation rate and mechanism. Another example is a starch-based polymer developed by Wang et al. [7]. They incorporated nanoparticles into a frozen starch gel to create a new superhydrophobic adsorbent. This material exhibited unique properties, forming a robust hydrophobic layer on the surface, enhancing adsorption capacity and stability, and expanding its application range, especially in oil removal and environmental protection. Corn starch-based polymers have been widely studied as well. They can be processed into films with good mechanical properties and biodegradability. Potato starch-based polymers are often used in food packaging due to their high water absorption capacity and ability to form a stable gel structure. Cassava starch-based polymers show unique properties in terms of flexibility and heat resistance, making them suitable for certain industrial applications. These polymers can be synthesized by incorporating starch with other substances or through chemical modifications of starch. They possess good biodegradability due to the presence of starch, which can be broken down by enzymes such as amylase and glucosidase in the environment [35].

The growing enthusiasm for bio-based composite materials has propelled a surge in research on starch-based polymers [2]. For example, recent studies have explored the use of nanoparticles and specific chemical modifications to enhance the mechanical properties and stability of starch. Wang et al. [7] introduced an innovative approach by incorporating nanoparticles into a frozen starch gel to create a new superhydrophobic adsorbent. This material exhibited unique properties and formed a robust hydrophobic layer on the surface of the adsorbent, thereby enhancing its adsorption capacity and stability and ultimately expanding its application range. Such advancements have enabled starch-based polymers to be utilized in a wide array of applications, including food packaging, environmental remediation, and biomedical fields [48]. Starch-based polymers possess excellent biodegradability, which renders them environmentally friendly with negligible negative impacts [49]. A recent study by Qiu et al. [50] demonstrated that exogenous enzymes, such as amylase and protease, can efficiently promote the enzymatic degradation of starch-based polymers. The degradation process mainly involves the cleavage of the α-1,4-glycosidic linkages in starch-based polymers by enzymes like amylase and glucosidase [35]. Environmental factors, such as temperatures in the range of 30 °C to 60 °C and humidity above 80%, can significantly accelerate this process [51]. These polymers primarily degrade via two pathways: (i) physical breakdown and (ii) biological breakdown [51]. In a research by Tetlow et al. [52], it was reported that the modification of starch with specific chemical groups can enhance its resistance to physical breakdown and improve its overall stability. Additionally, the study by Pfister et al. [53] showed that the combination of starch with other polymers can result in materials with improved mechanical and degradation properties. For example, the blend of starch and polyvinyl alcohol exhibited enhanced tensile strength and controlled degradation behavior. Regarding the physical breakdown of starch-based polymers, it is highly temperature-dependent. In the temperature range of 50–70 °C and humidity levels of 60–80%, physical changes such as swelling and moisture absorption are more prominent. At around 50 °C, starch polymers start to show significant swelling due to increased molecular mobility and interaction with water molecules. As the temperature approaches 70 °C, the rate of moisture absorption accelerates, which may lead to mechanical failure as the polymer structure becomes distorted [54].Cross-linking can also occur at elevated temperatures, typically above 80 °C. This cross-linking process alters the polymer’s chemical structure and often results in an accelerated degradation rate [55]. For example, in some starch-based films, when heated above 80 °C, the formation of cross-links between starch chains was observed, which made the films more brittle and prone to degradation. In terms of biological breakdown, environmental factors play a crucial role. The activity of amylase and other enzymes is optimal in a relatively narrow temperature range. Between 30 °C and 50 °C, the enzymatic degradation of starch-based polymers is most efficient. At lower temperatures, the enzymatic activity decreases, slowing down the degradation process. At temperatures above 50 °C, although the overall reaction rate may increase due to enhanced molecular motion, the enzymes may start to denature, losing their catalytic activity and thereby affecting the degradation kinetics. Humidity also has a significant impact. When the humidity is above 80%, it provides a favorable environment for enzyme activity and microbial growth, which in turn accelerates the biological breakdown of starch-based polymers. Microorganisms present in the environment can secrete a variety of enzymes that specifically target the α-1,4-glycosidic linkages in starch, breaking the polymer chains into smaller fragments that can be further metabolized.

Starch-based polymers tend to swell and absorb moisture, particularly in humid environments, which may induce mechanical failure owing to size variations [54]. Starch-based polymers are also known to undergo cross-linking at elevated temperatures, resulting in an accelerated degradation rate [55]. Conversely, in terms of biological breakdown, microorganisms and enzymes present in both biological and non-biological environments play key roles [56]. During this breakdown, starch-based polymers are disintegrated into sugar-like molecules, which are then absorbed and utilized by the microorganisms. These degradation processes extensively modify the properties and structures of the polymers. In a recent study, Qiu et al. [57] found that exogenous enzymes, such as amylase and protease, can efficiently promote the enzymatic degradation of starch-based polymers. For example, corn starch-based polymers have been widely studied. They can be processed into films with good mechanical properties and biodegradability. Potato starch-based polymers, on the other hand, are often used in food packaging due to their high water absorption capacity and ability to form a stable gel structure. Cassava starch-based polymers show unique properties in terms of flexibility and heat resistance, making them suitable for certain industrial applications.

The primary forces governing the interactions among molecular chains in starch are hydrogen bonding and van der Waals forces, both of which are influenced by factors such as temperature, pressure, and solution concentration [50]. Changes in external conditions can disrupt the thermodynamic equilibrium, impairing the balance of these forces and leading to structural changes in the material. In the solution state, an increase in temperature or a decrease in solution concentration weakens the hydrogen bonding interactions among molecular chains in starch, resulting in depolymerization, granule disruption, and, ultimately, material dissolution. High temperatures (50–70 °C) also weaken the van der Waals forces (intermolecular attractions) among individual molecular chains in starch, ultimately leading to the breakdown of starch materials.

Starch is among the most prevalent polysaccharides in nature. It is primarily composed of two types of molecules: (i) linear amylose, formed by the direct connection of numerous glucose molecules via α-1,4-glycosidic bonds, and (ii) branched amylopectin, which also contains α-1,6-glycosidic bonds [58,59]. Owing to these two structural forms, starch behaves differently under different environmental conditions. In starch, amylopectin and amylose are organized into semi-crystalline and amorphous layers arranged in the form of concentric rings [60]. This layered structure allows starch to store and release energy efficiently. Given that amylopectin features more numerous branches than amylose, it exhibits higher solubility and moisture absorption capacity. These attributes make amylopectin more susceptible to enzyme-induced degradation, releasing energy for organisms. In contrast, amylose is more resistant to such degradation, requiring more time and enzymes for digestion.

The differing structures of amylopectin and amylose substantially impact the digestion and absorption of starch in food. The different structures of amylopectin and amylose in starch lead to varying digestion and absorption rates of different foods, as detailed in [61].

When amylose interacts with iodine and certain organic reagents (including specific organic reagents, such as methanol and ethanol, which are used to facilitate the breakdown of polymer chains under controlled conditions), it forms complexes exhibiting distinctive properties in solution [28,29]. The formation of these complexes is indicated through color changes, such as from colorless to blue or black. This property enables the widespread use of amylose in chemical experiments and other applications.

**Figure 4:**
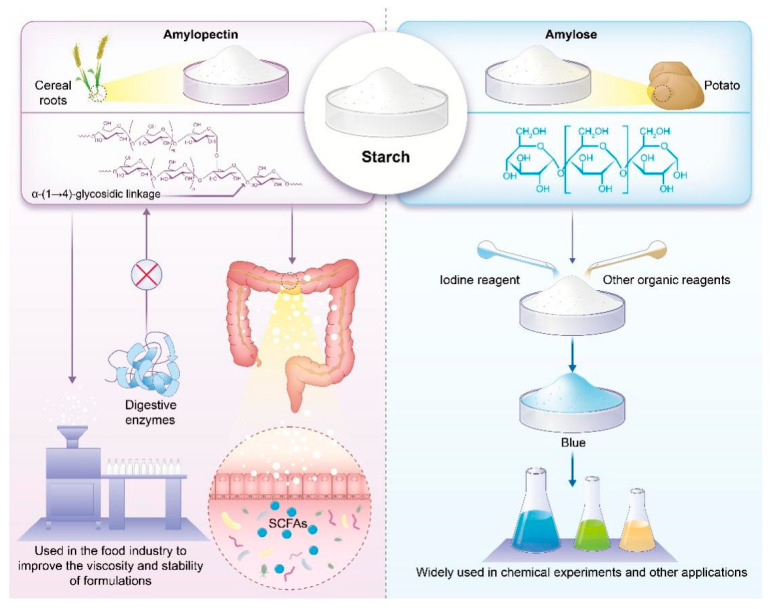
Structural characteristics and applications of amylose and amylopectin in starch-based materials [35].

The structural characteristics and applications of amylose and amylopectin in starch-based materials are shown in Figure 4. Amylopectin chains are bonded to the primary amylose molecule via *α-1*,4-glycosidic linkages [62]. Owing to the presence of these chains, branched starch exhibits greater solubility and gelatinization ability than straight-chain starch, making it a preferred choice in the food industry for improving the viscosity and stability of formulations. Additionally, branched starch exhibits specific physiological functions. Owing to its branched structure, which is resistant to degradation by digestive enzymes, branched starch can act as a fiber with prebiotic properties [1,2,3]. It can undergo fermentation in the colon, producing short-chain fatty acids that provide energy to intestinal bacteria. Hence, understanding the structure and properties of branched starch can facilitate the exploitation of its excellent physicochemical properties and physiological functions in food and health applications.

**Figure 5:**
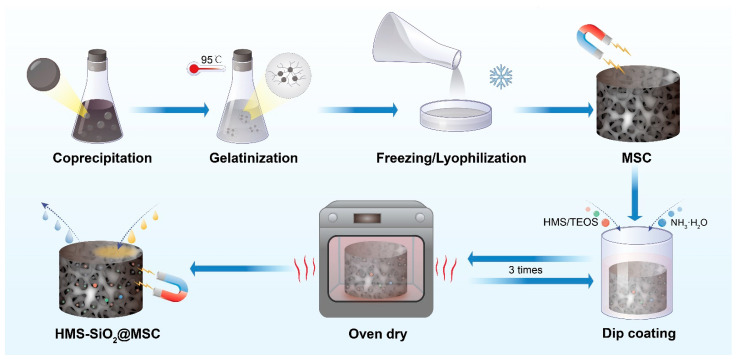
Schematic of the fabrication of a superhydrophobic adsorbent.

Wang et al. [7] introduced an innovative approach by incorporating nanoparticles into a frozen starch gel to create a new superhydrophobic adsorbent (Figure 5). This material was found to exhibit unique properties and formed a robust hydrophobic layer on the surface of the adsorbent, thereby enhancing its adsorption capacity and stability and ultimately expanding its application range. This study made a notable contribution to addressing environmental pollution and wastewater treatment challenges while offering novel insights and directions for material development and design. Notably, superhydrophobic adsorbents demonstrate exceptional oil adsorption capacity, which stems from several key factors. First, the chemical stability provided by their covalent bonds (Si–O–C/Si) enhances the hydrophobic properties of oily substances. When such oil-based substances contact the adsorbent surface, they are more likely to interact with the adsorbent than with water molecules in the surrounding environment [63]. Second, the pore walls of these superhydrophobic adsorbents play a crucial role in providing support and enhancing their ability to capture and retain oily substances. This structural framework also creates a barrier that prevents air ingress, further increasing the oil adsorption capacity [64,65,66]. Consequently, superhydrophobic adsorbents possess robust oil absorption functionality, making them suitable for applications in fields such as oil pollution treatment and environmental protection.

Figure 5 presents the schematic of fabricating a superhydrophobic adsorbent by incorporating nanoparticles into a frozen starch gel. The unique properties of this adsorbent are mainly attributed to several factors. Firstly, the nanoparticles contribute to the formation of a robust hydrophobic layer on the surface. This layer is formed through a combination of the chemical properties of the nanoparticles and their interaction with the starch gel. The nanoparticles can modify the surface energy of the adsorbent, making it more favorable for the adsorption of hydrophobic substances like oils. Secondly, the structure of the frozen starch gel itself plays a role. The gel provides a porous matrix that allows the nanoparticles to be dispersed evenly and also provides a large surface area for adsorption. When the adsorbent comes into contact with an oil-water mixture, the hydrophobic layer repels water while attracting and adsorbing oil molecules. The pore walls of the adsorbent help to capture and retain the oil, preventing it from being released. Additionally, the chemical stability of the covalent bonds (Si–O–C/Si) in the adsorbent enhances its hydrophobic properties. This stability ensures that the adsorbent can maintain its performance even in harsh environmental conditions. The superhydrophobic adsorbent has wide applications in oil pollution treatment and environmental protection. It can be used to clean up oil spills in water bodies, where it can efficiently adsorb and remove the oil, reducing the environmental impact. It can also be applied in industrial wastewater treatment to remove oil and other hydrophobic contaminants, improving the quality of the treated water.

### 2.4. Plant Fiber-Based Polymers

Plant fiber-based polymers, as defined in this review, are polymers that contain a significant amount of plant fibers (not necessarily over 50%) and derive their properties and biodegradation characteristics from the presence of these fibers. These polymers can be synthesized through various methods, such as microbial fermentation of plant fibers or chemical treatment of cellulose with acrylic monomers. Islam et al. [67] reviewed the processing, properties, and applications of green composites from natural fibers and biopolymers, which provides valuable insights into the understanding of plant fiber-based polymers.

The degradation mechanism of plant fiber-based polymers is mainly through the biodegradation process of its cellulose component. In the natural environment, cellulose is broken down by cellulase in fungi and bacteria. This process is significantly influenced by temperature (the best range is 50 °C to 70 °C) and humidity (better than 85%), conditions that promote microbial activity and the efficiency of enzyme action [68].

**Figure 6:**
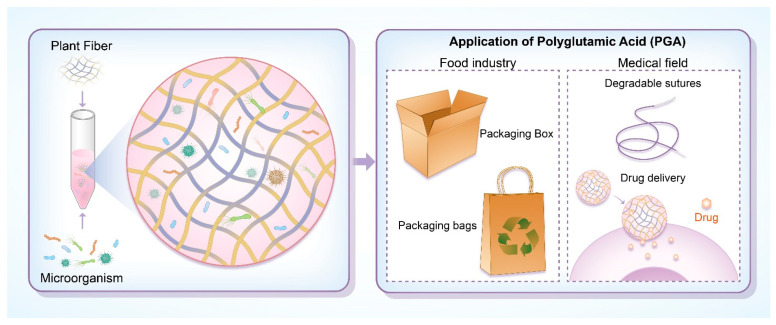
Application of PGA.

Polyglutamic acid (PGA) is an eco-friendly, biodegradable, and renewable substance produced through the microbial fermentation of plant fibers, with applications in environmental protection, food packaging, and the pharmaceutical field. For example, a recent study focused on the optimization of the PGA production process and found that by adjusting the fermentation conditions, the yield and quality of PGA could be significantly improved. The specific changes in fermentation parameters and their impact on PGA properties were detailed in the study, providing a valuable reference for the industrial production of PGA [69]. Consequently, microorganisms can substitute fossil fuels in the production of PGA materials. As a naturally occurring substance, PGA can be absorbed by plants [70]. In the biomedical field, PGA is used in biodegradable sutures and drug delivery systems, as illustrated in Figure 6 [71].

**Figure 7:**
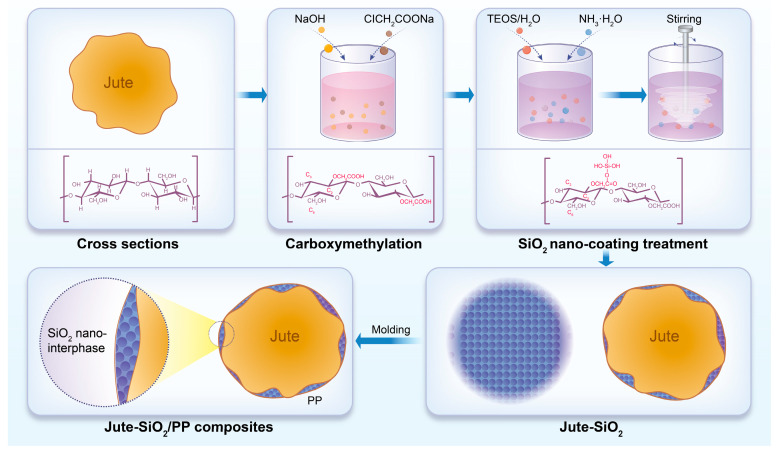
Schematic depicting the synthesis of jute/SiO_2_/PP composites using the sol–gel technique and compression molding.

Polyacrylic acid (PAA) can be synthesized through the chemical reaction of cellulose in natural fibers such as cotton, kapok, and bamboo with acrylic monomers. For example, in a study by Li [72], sugarcane bagasse, a cellulose-rich natural fiber, was used as the starting material. The bagasse was first treated with sodium hydroxide and bleached to separate the non-cellulosic components, obtaining cellulose (CE) fibers. Then, through a simple free-radical graft polymerization process, crosslinked cellulose-poly (acrylic acid sodium) hydrogels (CE-PAANA) were successfully synthesized for the removal of heavy metal ions. The experimental process was detailed, including the characterization of the hydrogel’s structure and morphology, as well as the investigation of various factors (such as pH, contact time, and solution concentration) that affect the batch adsorption capacity. The results showed that the adsorption kinetics fit well with the pseudo-second-order kinetic model, and the adsorption isotherm conformed to the Langmuir model. The maximum adsorption capacities for Cu (II), Pb (II), and Cd (II) were calculated to be 106.3, 333.3, and 163.9 mg/g, respectively, using the Langmuir model. In addition, the X-ray photoelectron spectroscopy (XPS) and energy-dispersive X-ray spectroscopy (EDS) results demonstrated that cation exchange and electrostatic interactions were the main adsorption mechanisms for heavy metal ions. This research demonstrated the potential application value of CE-PAANA graft copolymer adsorbents obtained from cellulose-rich sugarcane bagasse in the removal of heavy metal ions. These natural fibers are renowned for their high biodegradability and ability to decompose naturally in the environment. For instance, a study by [67] showed that cotton fiber-based PAA composites exhibited excellent mechanical properties and biodegradability, making them ideal candidates for biodegradable products such as food packaging materials, medical supplies, and disposable products.The fibers possess remarkable toughness and strength, which contribute to their suitability in these applications. Apart from microbial fermentation, PAA can be synthesized via the chemical reaction of cellulose in plant fibers with acrylic monomers [73]. A specific example is the research by the authors of [73], which detailed the reaction conditions and the resulting properties of PAA. Due to its distinct chemical and physical characteristics, PAA has found extensive applications in the paper, pulp, and textile industries. It can enhance the strength and durability of paper products, improve the quality of pulp, and impart unique properties to textile materials. Significantly, Abdul Azam et al. [74] carried out a detailed study that demonstrated the addition of multi-walled carbon nanotubes to PP composites containing PGA and/or PAA led to a substantial improvement in their water absorption capacity. The study systematically investigated the effect of different concentrations of nanotubes and the interaction mechanisms with the polymer matrix, providing valuable insights for the design and optimization of such composites.Huang et al. conducted a comprehensive review of the research progress of different plant fiber-reinforced PLA/PBAT/PBS biodegradable composites [75]. They analyzed how the fiber length and diameter affected the mechanical properties of the composites. For example, longer fibers generally enhance tensile strength but could also lead to processing difficulties if not properly dispersed. The fiber diameter influenced the surface area available for interaction with the polymer matrix, with smaller diameters providing more surface area and potentially stronger interfacial bonding. The study also explored the impact of fiber content on the thermal stability of the composites. Higher fiber content could increase the thermal degradation temperature in some cases due to the higher thermal stability of the fibers themselves. Additionally, the review examined the role of different surface treatments on the fibers in improving the interfacial adhesion with the polymer matrix. For instance, plasma treatment of plant fibers was found to increase the surface energy and roughness, promoting better wetting and adhesion with the polymer, which in turn enhanced the overall mechanical properties of the composites. This research provides valuable insights for optimizing the properties of plant fiber-based polymer composites and understanding their potential applications. Moreover, the MMT formed a nano-layered structure that lowered the permeability and the rate of water absorption. Liu et al. [37] blended PP, silica (SiO_2_), and jute fiber using molten gel molding (Figure 7). This technology enabled the precise shaping and sizing of environmentally friendly fiber-based composites, which demonstrated remarkable performance. Molecular dynamics simulations revealed an intriguing phenomenon of molecular chain entanglement between the jute fibers and PP, enhancing the interfacial stability and preventing water molecules from penetrating the composite, thereby extending its service life. This finding is particularly significant, as it supports the development of more durable composite materials and offers valuable insights for future research and applications.

The water absorption performance of composite materials is influenced by the types of polymers and plant fibers, fiber treatments, rates of water absorption, and levels of environmental humidity [37]. Therefore, these factors must be carefully considered when addressing water absorption issues. Specifically, the water uptake performance of different polymers and plant fibers varies. Consequently, their appropriate selection during composite synthesis is vital for enhancing water absorption performance [76]. However, as plant fibers are inherently hydrophilic, increasing their concentration may enhance their water absorption capacity but negatively impact their mechanical properties [77,78]. Studies indicate that the water absorption capacity of plant fibers can be moderated by treating them either chemically or physically to alter their surface characteristics [79]. Additionally, environmental moisture levels and water absorption characteristics can be adjusted to improve overall moisture uptake performance. Research also reveals that synthesis processes and parameter selection can significantly enhance the water uptake capacity of composite materials [80]. Therefore, further explorations into the possibility of combining polymer coating methods with physical or chemical treatments may offer a promising approach for improving the moisture stability of plant fiber composites.

Polymers are well regarded for their resistance to rain- and humidity-induced water damage [81]; however, in some cases, this resistance can be further boosted. For instance, different coating methods can alter the surface morphologies and chemical properties of composite materials, enhancing their waterproofing capabilities [82]. Recent advancements have shown that nano-enhanced coatings can dramatically improve the water resistance of plant fiber-based composites, making them more suitable for outdoor applications [83]. Studies indicate that modifications in coating formulations may improve moisture resistance by adjusting surface structure and chemical properties [84]. By integrating these coating methods with complementary technologies, we can not only enhance the waterproofing performance of plant fiber composites but also enable them to maintain durability in humid environments over extended periods.

For instance, plasma treatment is known to increase the surface energy and chemical activity of plant fibers, promoting the formation of active or functional groups on their surfaces [81]. This enhancement increases the effectiveness of coatings applied to plasma-treated fibers, thereby improving the adhesion between the two surfaces. Additionally, incorporating nanoparticles into coatings can further enhance their water resistance [76]. The increased surface area provided by the nanoparticles creates more contact points, strengthening the bonding between the coating material and the water molecules and thus improving waterproofing performance [77]. Thus, in conclusion, polymer coating technology, combined with other physical or chemical processes, can be used to enhance the water resistance of plant fiber-based composite materials. This treatment results in the formation of a polymer protective film on the surface of the composite, preventing the ingress of water molecules. Moreover, the coating layer can be tailored to meet the specific requirements of the application environment, thereby enhancing the waterproofing performance of the composite [78].

In addition to plasma treatment, plant fibers can also be subjected to electron beam irradiation, which strengthens the bond between the fibers and coatings, thereby enhancing stability and waterproofing performance [79]. The addition of nanoparticles to the composite formulation can further improve waterproofing performance by raising the interfacial energy [80]. Meanwhile, adding a crosslinking agent can create a composite structure that promotes strong adhesion between the coating and plant fibers, ensuring effective waterproofing [80]. Thus, combining polymer coating technology with other physical or chemical techniques offers promising potential for enhancing the waterproofing performance of plant fiber-based composites and broadens their application range.

In order to more clearly compare the characteristics of different types of biodegradable polymers, Table 2 summarizes their typical examples, degradation conditions and specific degradation observation results.

## 3. Conclusions

In conclusion, this review not only explored the synthesis routes and degradation pathways of biodegradable polymers but also provided a critical summary of the reaction conditions and mechanisms. Understanding these aspects is essential for advancing the development of environmentally sustainable biodegradable polymers. This review explored the synthesis routes of biodegradable polymers and their degradation pathways in natural environments. For instance, biodegradable materials, such as carbohydrates are broken down by microorganisms. These organisms use enzymes to cleave large polymers into smaller units, which are then absorbed and metabolized. The factors influencing the rate of biodegradation include temperature, humidity, and oxygen availability. Higher temperatures and humidity levels generally accelerate decomposition, whereas low oxygen levels may slow it down. Additionally, given that polymers vary in their chemical composition and physical characteristics, the selection of biodegradable materials should align with their intended application. Understanding the specific degradation mechanisms of biodegradable polymers not only supports their application in environmentally sensitive areas but also helps to design polymers with a predicted lifetime to suit specific application needs. The degradation products of these polymers should be non-toxic substances that can be safely broken down in the natural environment. Notably, polylactic acid (PLA) composites incorporating hydroxyapatite (HA) have demonstrated enhanced mechanical properties and biocompatibility, making them highly suitable for biomedical applications, such as drug delivery systems. Similarly, starch-based polymers, when modified with nanoparticles, exhibit superhydrophobic properties, significantly improving their performance in environmental applications like oil spill cleanup. These advancements highlight the potential of biodegradable polymers to address critical challenges in sustainability and environmental protection. A comprehensive understanding of polymer degradation mechanisms not only provides essential insights into their structural changes over time but also guides the selection of physical properties and chemical compositions that are best suited for specific applications. For instance, the selection process involves determining the required mechanical strength, thermal stability, and biodegradability of the polymer based on its application in medical devices, agricultural films, or packaging materials. This tailored approach ensures that each polymer’s properties align with its environmental and functional demands, thereby enhancing its performance and sustainability in targeted applications. Polymers and their composites synthesized based on this understanding can offer versatile applications while promoting sustainability and a positive environmental impact.

## Figures and Tables

**Table 1:** Experimental conditions for synthesis and degradation of biodegradable polymers.

Polymer Type	Synthesis Conditions	Degradation Conditions	Key Observations
Polylactic Acid (PLA)	Lactic acid is polymerized under controlled temperatures (140–180 °C) with catalysts like Sn(Oct)_2_, SnCl_4_, and other metal catalysts used [30].	In a microbial environment (with Bacillus, Pseudomonas, Stenotrophomonas, etc.), at temperatures (30–60 °C), or under enzyme (Esterase, keratinase, lipase, and protease, etc.) conditions [31].	HA in PLA composites promotes cold crystallization, enhancing degradation. Ion exchange between PLA and β-TCP in SBF leads to surface reactions, forming a Ca–P framework. Crystalline regions due to HA are more prone to hydrolysis and enzymatic attack [32].
Starch-Based Polymers	Processed with plasticizer (Glycerin, sorbose, etc.) or treated with heat-moisture (90–180 °C) [33].	Degrades in humid conditions (>60% RH) with enzymatic activity by amylase and glucosidase at 30–50 °C [34].	Enzymatic treatment speeds up polymer breakdown into glucose. Corn starch polymers form films with good properties. Potato starch polymers are used in food packaging for water absorption and gel formation. Cassava starch polymers have flexibility and heat resistance for industrial use [35].
Plant Fiber-Based Polymers	Through microbial fermentation or chemical treatment of cellulose with acrylic monomers at temperatures of 60–80 °C [36].	In the compound microflora (WSC-9, etc.), when the solution conditions change, the temperature is about 50 °C. The specific microorganisms in the WSC-9 microflora have optimal enzymatic activities at around this temperature, thereby playing a significant role in breaking down the polymers.	Nanoparticles and surface modification reduce water absorption and enhance durability. Jute/SiO_2_/PP composites use molten gel molding. Molecular chain entanglement between jute and PP improves interfacial stability and reduces water penetration, extending service life [37].

**Table 2:** Examples and degradation details of biodegradable polymers.

**Polymer Group**	**Example**	**Degradation Conditions**	**Specific Degradation Observations**
PLA	PLA with HA composite (e.g., as studied by Omigbodun et al. [39])	In a microbial environment (with Bacillus, Pseudomonas, Stenotrophomonas, etc.), at temperatures (30–60 °C), or under enzyme (Esterase, keratinase, lipase, and protease, etc.) conditions. In SBF, the presence of HA promotes cold crystallization, which enhances degradation.	The ion exchange between PLA and β-TCP particles in SBF leads to surface recombination and dissolution reactions, forming a loose Ca–P framework. The crystalline regions formed due to HA presence are more susceptible to hydrolysis and enzymatic attack. The hydrolysis rate of PLA is affected by temperature and humidity, and the addition of catalysts like SnCl_2_ can enhance the process (as reported by Zaaba and Jaafar [38]). Photodegradation can also occur upon exposure to ultraviolet light.
Starch-based polymers	Corn starch-based polymers (as in the research of Patel et al. [33])	Degrades in humid conditions (>60% RH) with enzymatic activity by amylase and glucosidase at 30–50 °C. It can also degrade physically at 50–70 °C and humidity levels of 60–80% (as reported by Liu and Zhang [34]).	Enzymatic treatment accelerates polymer disintegration into glucose. Physical breakdown includes swelling, moisture absorption, and cross-linking at elevated temperatures. The degradation is mainly through the cleavage of α-1,4-glycosidic linkages. MaterBi (a starch-based material) is synthesized by blending starch with other biodegradable polymers and additives, and it exhibits good biodegradability and mechanical properties, degrading mainly through the enzymatic hydrolysis of starch by amylase and other enzymes present in the environment (as studied by Aldas et al. [47]).
Plant fiber-based polymers	Jute/SiO_2_/PP composites (studied by Liu et al. [37])	In the compound microflora (WSC-9, etc.), when the solution conditions change, the temperature is about 50 °C.	Molecular chain entanglement between jute fibers and PP enhances interfacial stability and reduces water absorption. Cellulose in jute is degraded by cellulase in fungi and bacteria, influenced by temperature and humidity. The water absorption performance of the composites is affected by the types of polymers and plant fibers, fiber treatments, rates of water absorption, and levels of environmental humidity. Plasma treatment of plant fibers increases surface energy and chemical activity, promoting the formation of active or functional groups on fiber surfaces and enhancing the adhesion between fibers and coatings (as shown by Macedo et al. [81]).

## Data Availability

The original contributions presented in the study are included in the article, further inquiries can be directed to the corresponding authors.
